# Supplementary material for: Influence of the Charge Ratio of Guanine-Quadruplex Structure-Based CpG Oligodeoxynucleotides and Cationic DOTAP Liposomes on Cytokine Induction Profiles
Source: Biomolecules. 2023 Nov 11;13(11):1639. doi: 10.3390/biom13111639 (PMC10669863; doi:10.3390/biom13111639)
Supplement: Supplementary file 1 [file biomolecules-13-01639-s001.zip › biomolecules-2613451-supplementary.pdf]

Supporting Information

Influence of the Charge Ratio of Guanine-Quadruplex Structure-Based CpG Oligodeoxynucleotides and Cationic DOTAP Liposomes on Cytokine Induction Profiles

Nguyen Bui Thao Le [1, 2], Anh Thi Tram Tu [1, 3, 4], Dandan Zhao [1], Chiaki Yoshikawa [1, 2], Kohsaku Kawakami[1], Yoshihisa Kaizuka[1], Tomohiko Yamazaki [1, 2*]

[1] Research Center for Macromolecules and Biomaterials, National Institute for Materials Science (NIMS), 1-2-1 Sengen, Tsukuba 305-0047, Japan; le.buithaonguyen@nims.go.jp (N.B.T.L.); tuthitramanh@gmail.com (A.T.T.T.); amberdiary@gmail.com (D.Z.); yoshikawa.chiaki@nims.go.jp (C.Y.); kawakami.kohsaku@nims.go.jp (K.K.); kaizuka.yoshihisa@nims.go.jp (Y.K.)

[2] Division of Life Science, Hokkaido University, Kita 10, Nishi 8, Kita-ku, Sapporo 060-0808, Japan

[3] Department of Magnetic and Biomedical Materials, Faculty of Materials Science and Technology, VNUHCM-University of Science, 227 Nguyen Van Cu street, Ward 4, District 5, Ho Chi Minh City 70000, Vietnam

[4] Ho Chi Minh City Campus, Vietnam National University, Linh Trung, Thu Duc, Ho Chi Minh City 70000, Vietnam

* Correspondence: yamazaki.tomohiko@nims.go.jp; Tel.: +81-29-859-2345; Fax: +81-29-859-2449

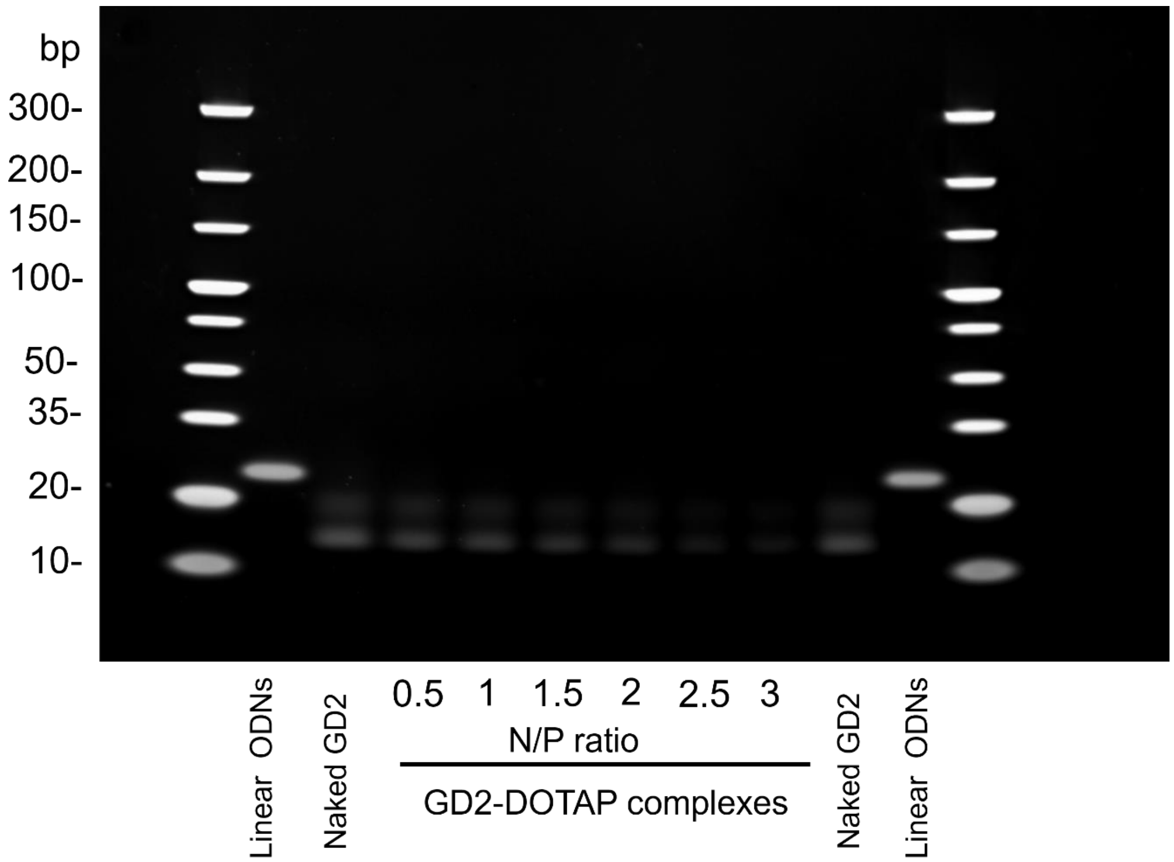

Figure S1. Polyacrylamide gel electrophoresis analysis of G4-CpG ODNs in complex with DOTAP. Electrophoresis was performed using a 10–20% linear gradient polyacrylamide gel in tris-glycine buffer supplemented with 4 mM KCl. Linear ODN is the ssODN with the same length (30mer) as GD2.

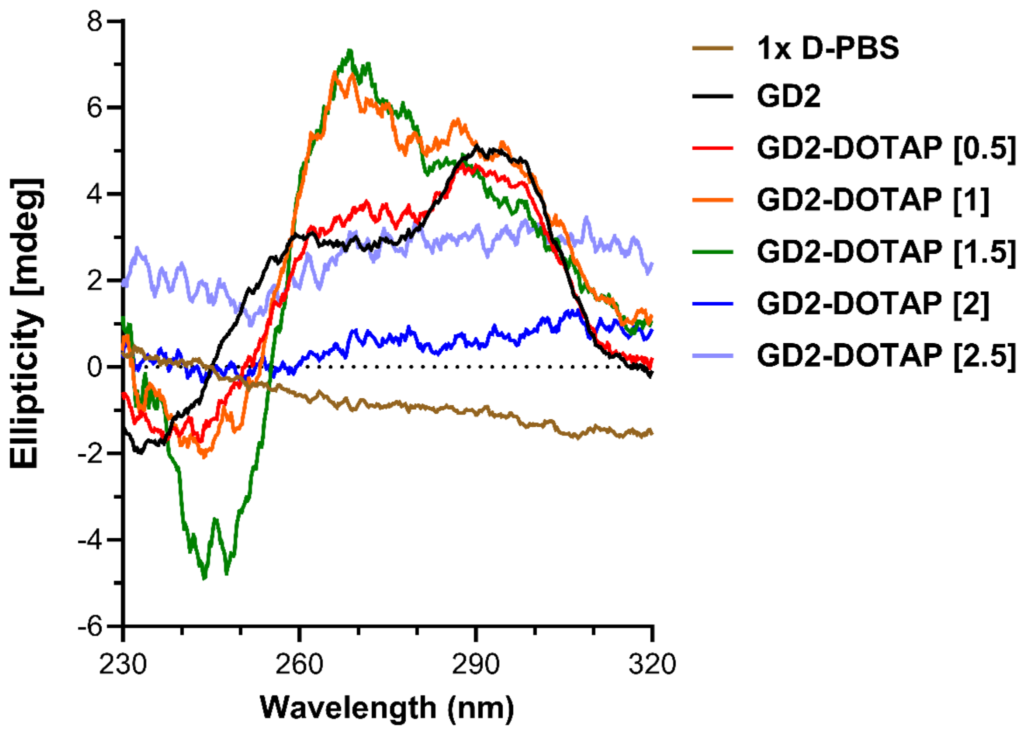

Figure S2. CD spectra of naked GD2 and GD2-DOTAP complexes were drawn from the original raw data.

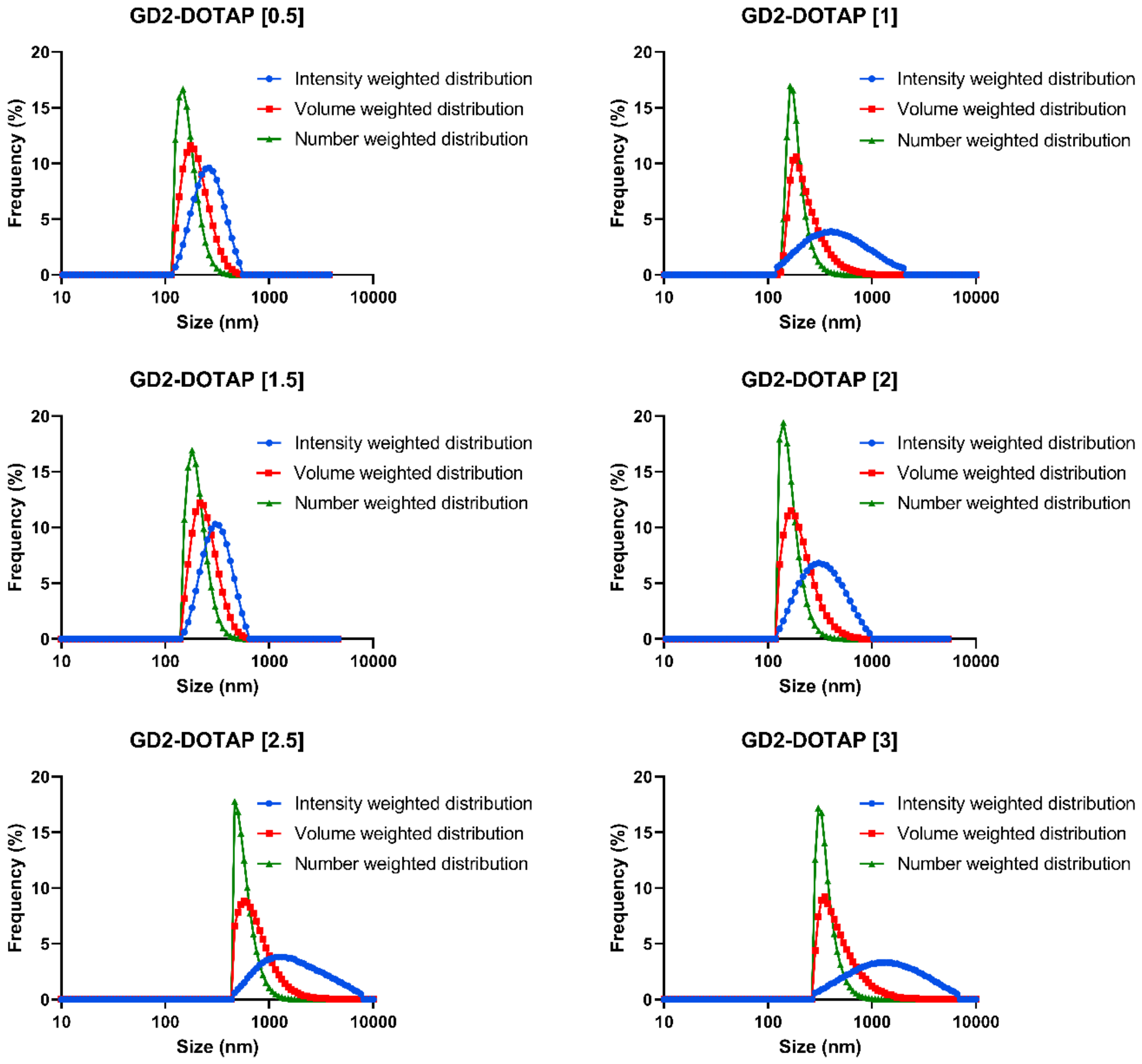

Figure S3. Intensity-, volume-, and number-weighted particle size distributions of GD2-DOTAP at various charge ratios. Distribution was calculated using the CONTIN algorithm.

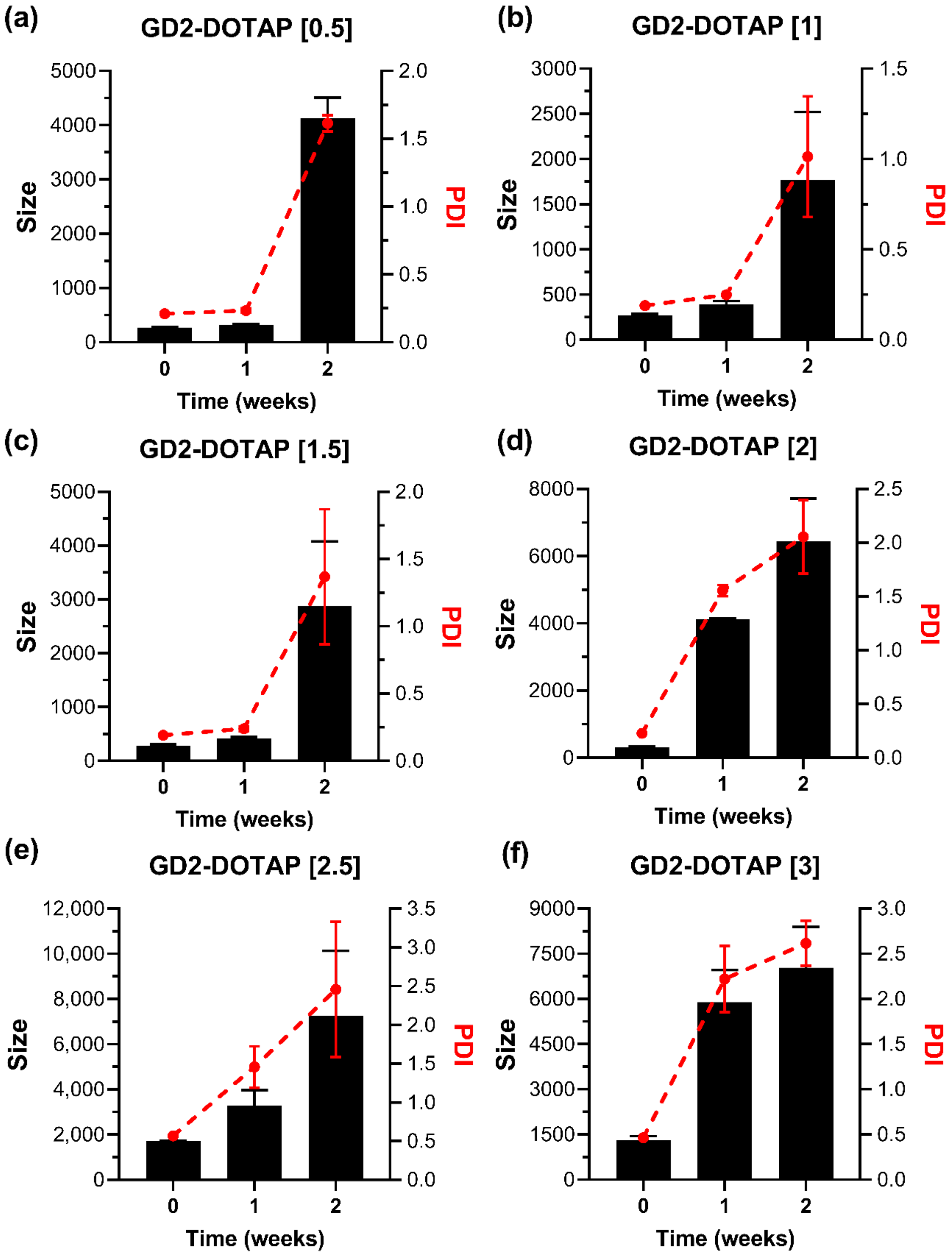

Figure S4. Hydrodynamic size (nm) reported as a function of time for all complexes at different charge ratios. Data are presented as mean ± SD (*n*=3).

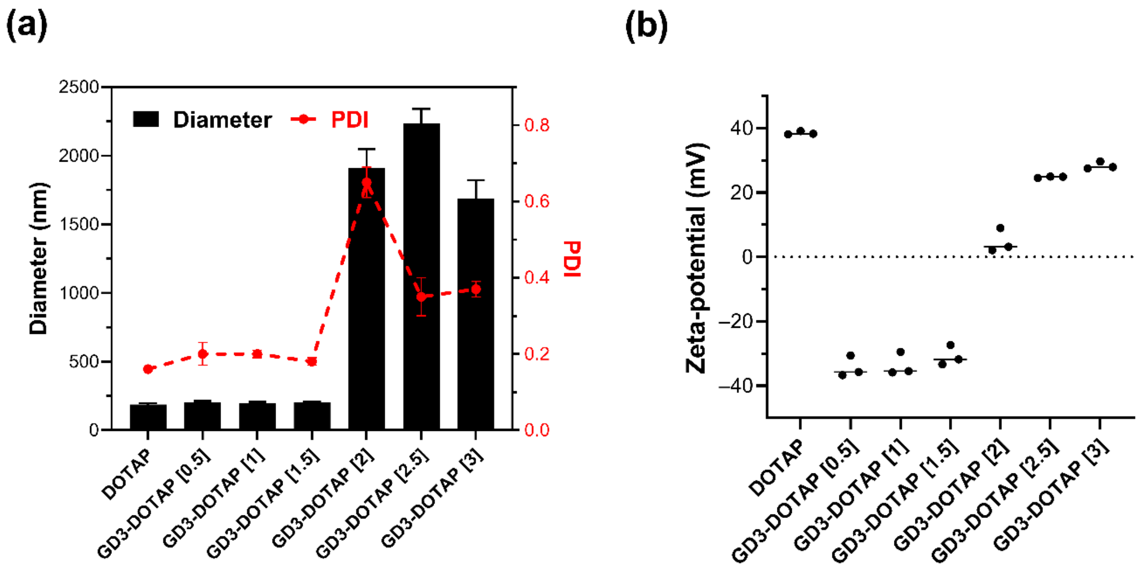

Figure S5. (**a**) Hydrodynamic size, polydispersity index, and (**b**) zeta potential of GD3-DOTAP complexes. Data are presented as mean ± SD (*n*=3).

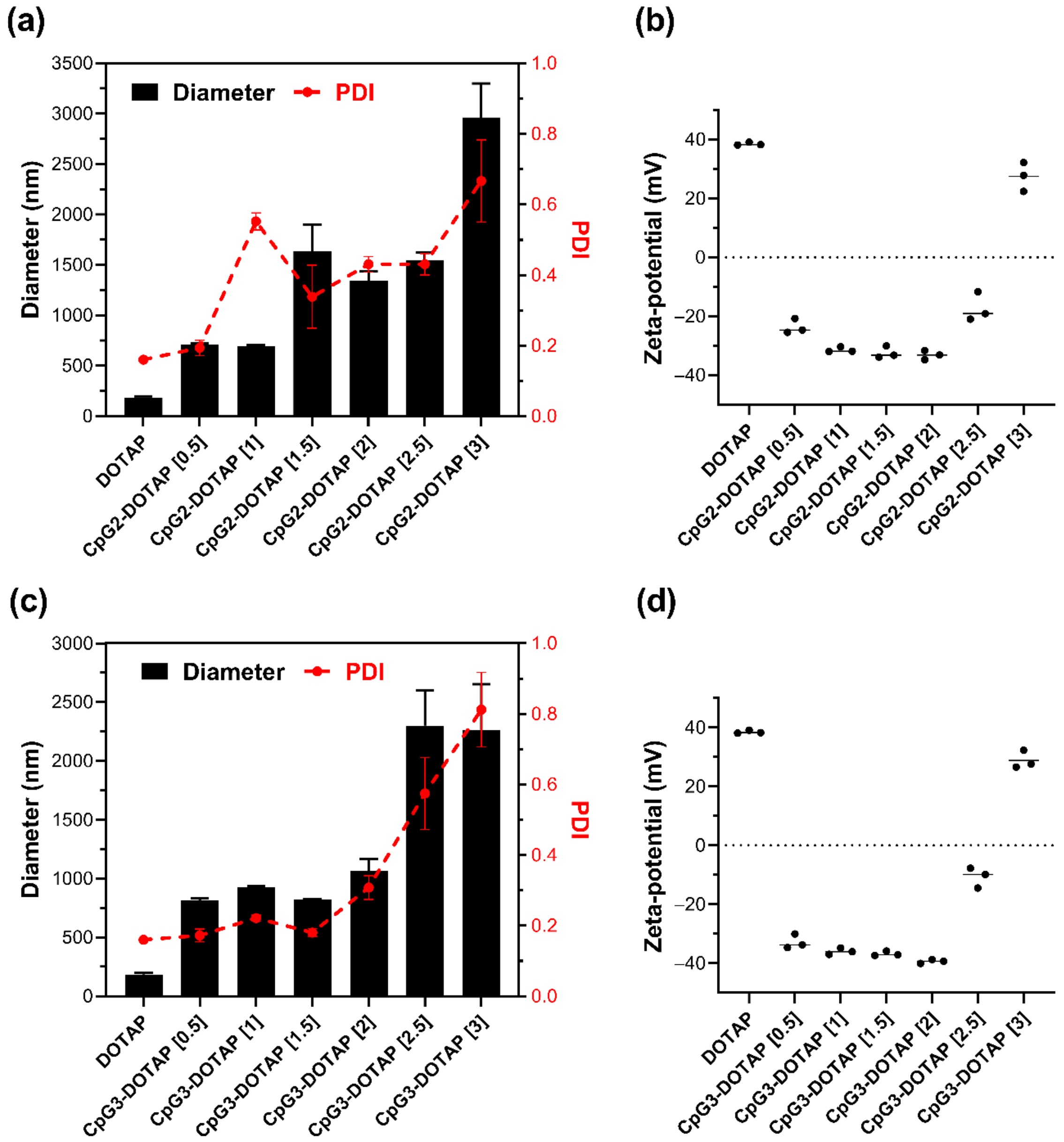

Figure S6. Characterization of linear CpG ODN-DOTAP liposome complexes of different charge ratios. (a, c) Hydrodynamic size, polydispersity index, and (b, d) Zeta potential. Data are presented as mean ± SD (*n*=3).

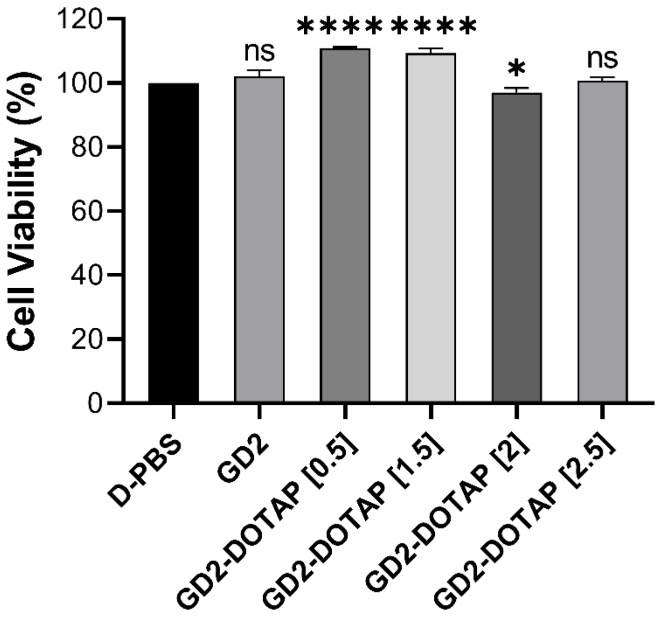

Figure S7. Cell viability of human PMBCs after stimulation with 0.5 μM Nacked GD2 and GD2-DOTAP for 48 h. Data are presented as mean ± SD (n=5). Statistical significance was calculated compared to non-treated cells (D-PBS). ****p<0.0001, *p<0.05, ns (not significantly different) p>0.05 (one-way analysis of variance, followed by Tukey's multiple comparisons test).

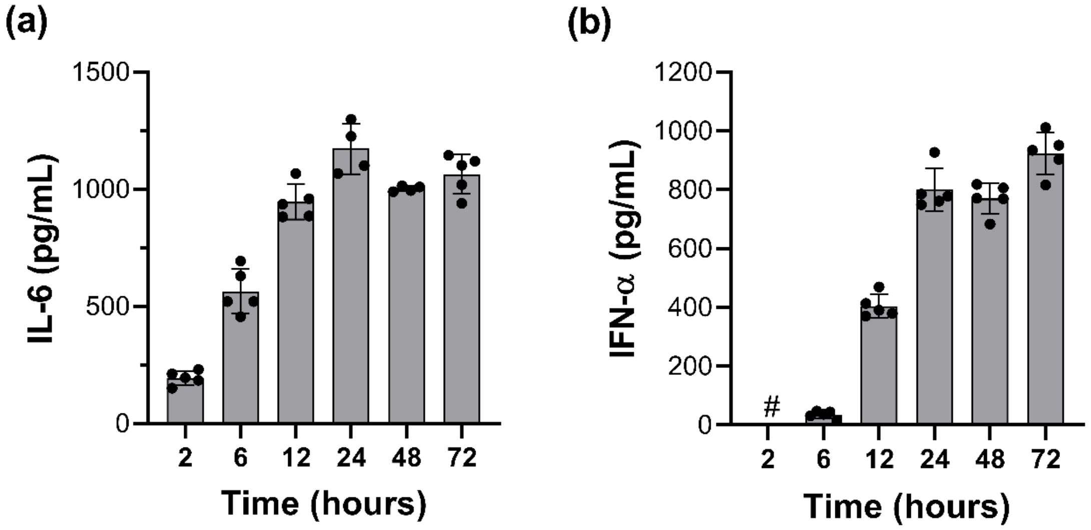

Figure S8. Cytokine induction by the GD2-DOTAP complex at a charge ratio of 1.5 in human PBMCs at different stimulation times. The final ODN concentration in the cell medium was 0.5 μM. Data are represented as mean ± SD (*n*=5). #, lower than the detection limit (3.9 pg/mL).

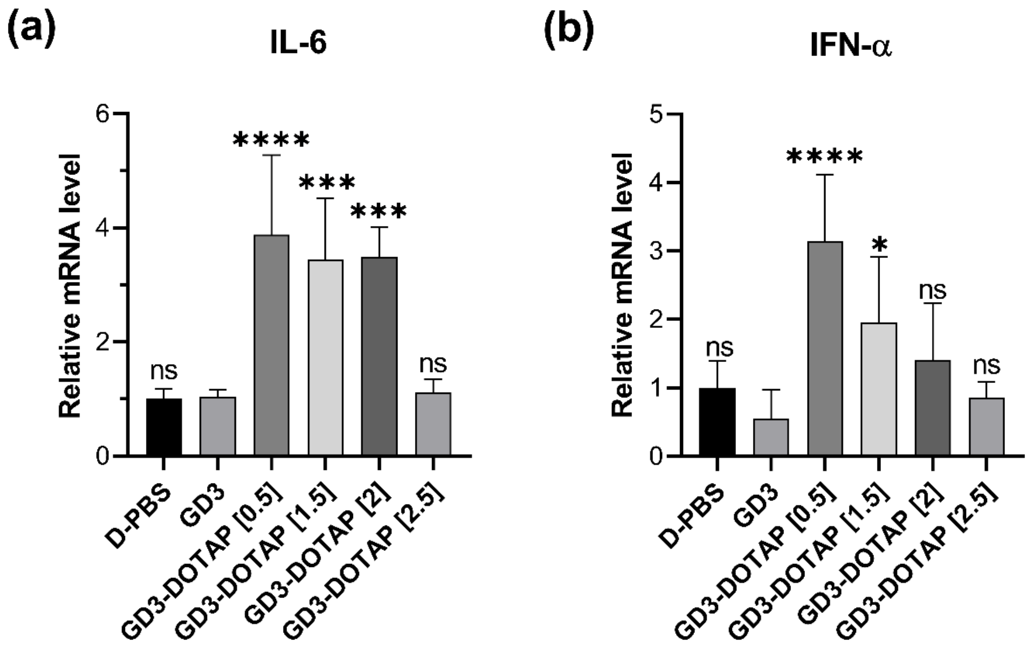

Figure S9. Cytokine induction by GD3-DOTAP complexes in (**a**) Namalwa and (**b**) PMDC05 cells. Relative mRNA levels of IL-6 and IFN-α in cells were examined after 6 h (in PMDC05 cells) and 4 h (in Namalwa cells) of stimulation with naked GD3 and GD3-DOTAP. The final ODNs concentration in the cell medium was 1 μM. Data are represented as mean ± SD (n=5). Statistical significance was calculated in comparison to bare GD3-treated cells (D-PBS). ****p<0.0001, ***p<0.001, *$p < 0.05$, ns (not significantly different) p>0.05 (one-way analysis of variance, followed by Tukey's multiple comparisons test).

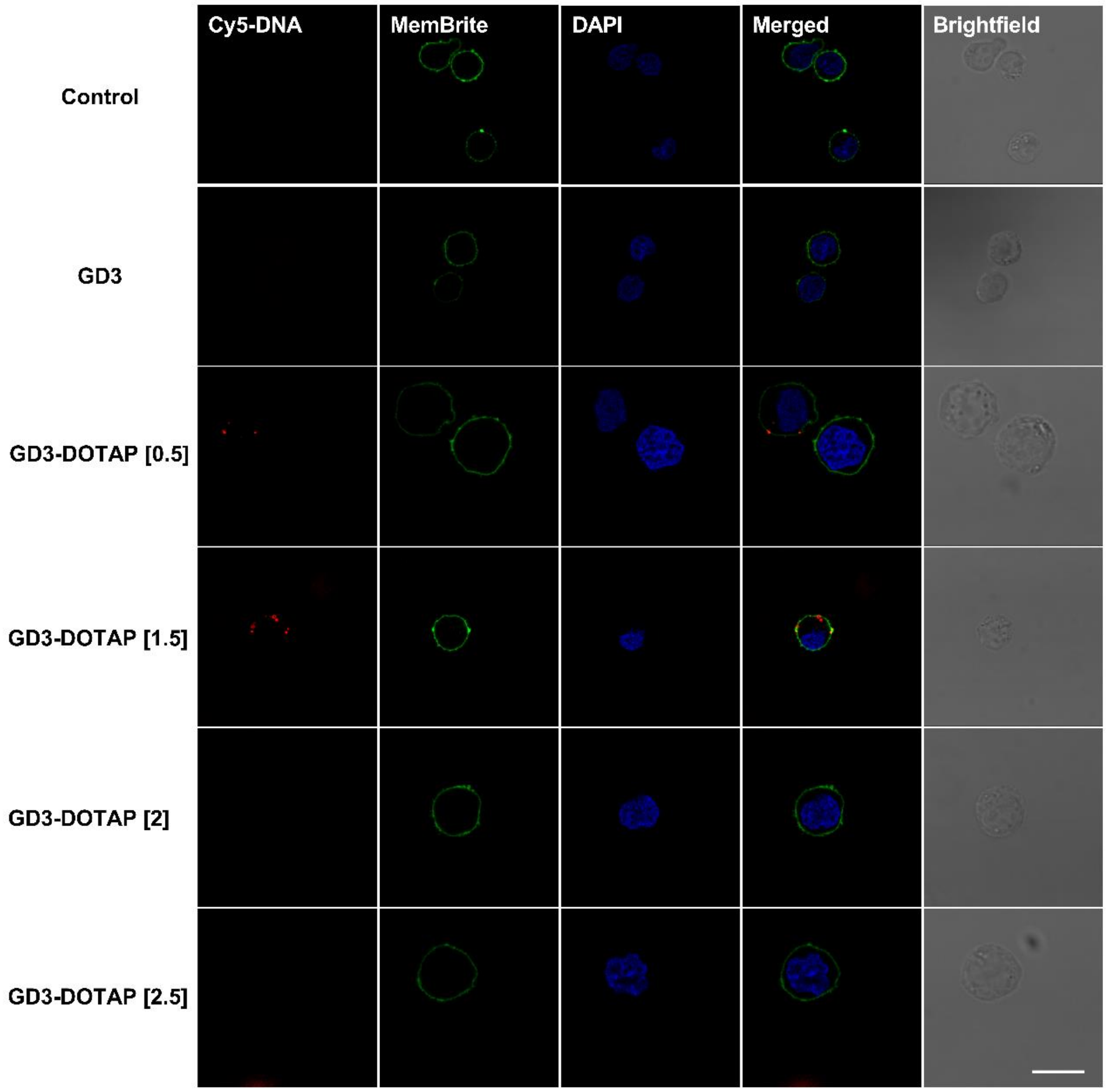

Figure S10. Internalization of naked GD3 and GD3-DOTAP complexes in Namalwa cells, after 2 h of stimulation. Non-treated cells served as the control. Cy5 (red), MemBrite™ (green), and DAPI (blue) represent GD3-DOTAP, cell membrane, and nuclei, respectively. Scale bar: 10 μm.

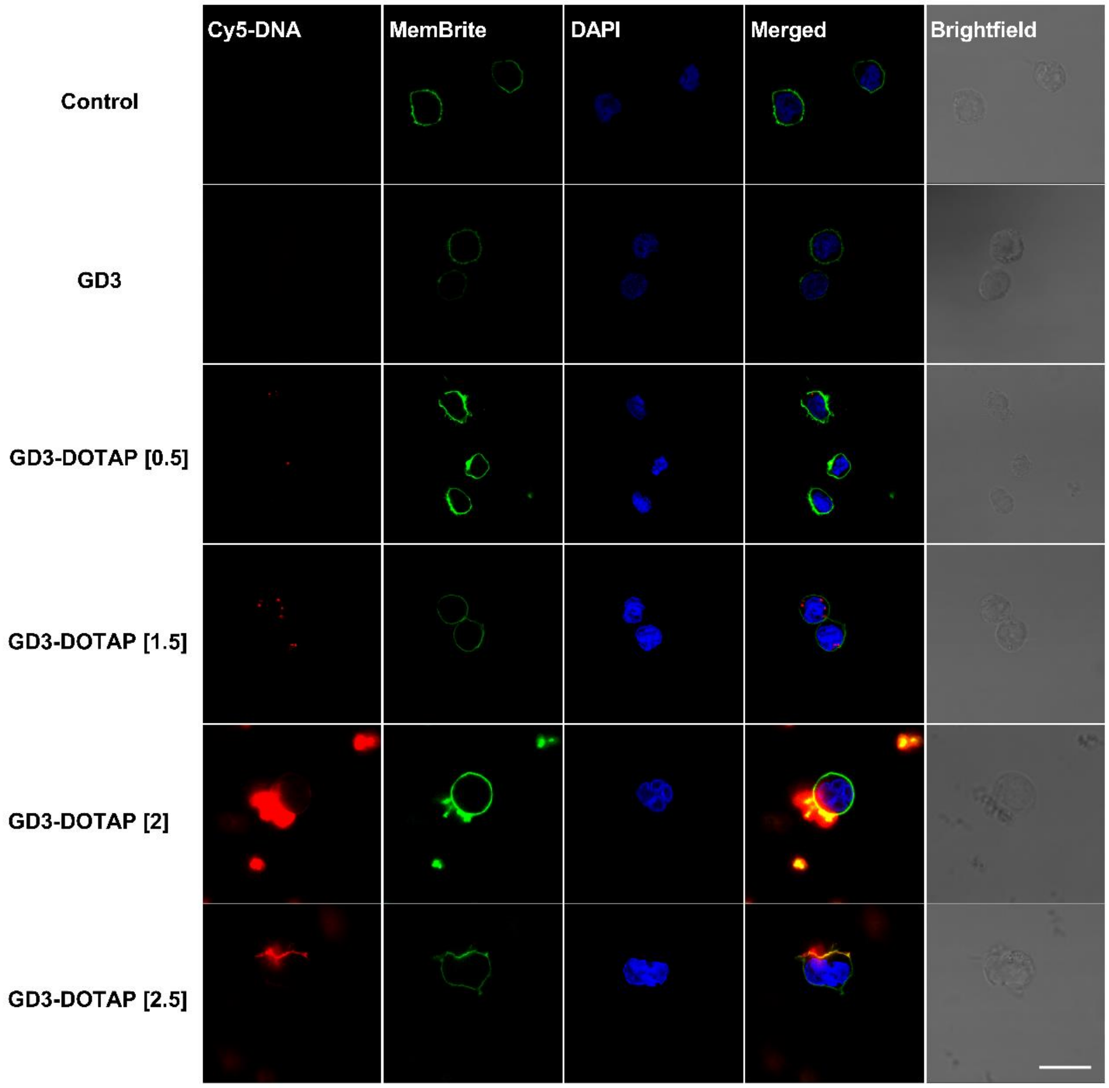

Figure S11. Internalization of naked GD3 and GD3-DOTAP complexes in PMDC05 cells, after 2 h of stimulation. The low-charge ratio complexes were localized inside the cells, while high-charge ratio complexes were still bound to the cell membrane. Non-treated cells served as the controls. Cy5 (red), MemBrite™ (green), and DAPI (blue) represent GD3-DOTAP, cell membrane, and nuclei, respectively. Scale bar: 10 μm.

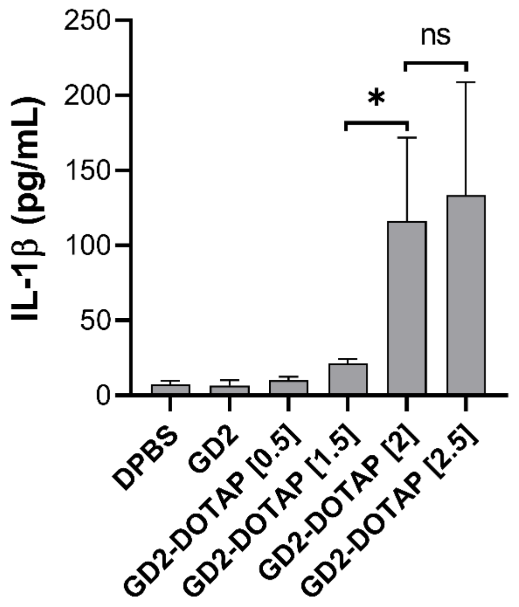

Figure S12. IL-1β production by GD2-DOTAP complexes in human PMBCs. The complexes at high-charge ratios (2 and 2.5) significantly induced IL-1β, which triggers the induction of pro-inflammatory cytokines, including IL-6. Data are represented as mean ± SD (n=5). *p<0.05, ns (not significantly different) p>0.05 (one-way analysis of variance, followed by Tukey's multiple comparisons test).
